# Microinjection of Antibodies Targeting the Lamin A/C Histone-Binding Site Blocks Mitotic Entry and Reveals Separate Chromatin Interactions with HP1, CenpB and PML

**DOI:** 10.3390/cells6020009

**Published:** 2017-03-25

**Authors:** Charles R. Dixon, Melpomeni Platani, Alexandr A. Makarov, Eric C. Schirmer

**Affiliations:** The Wellcome Trust Centre for Cell Biology, University of Edinburgh, Kings Buildings, Swann 5.22, Max Born Crescent, Edinburgh EH9 3BF, UK; s1581423@sms.ed.ac.uk (C.R.D.); m.platani@ed.ac.uk (M.P.); amcarov@gmail.com (A.A.M.)

**Keywords:** lamin, cell cycle, genome stability, DNA replication, centromere proteins

## Abstract

Lamins form a scaffold lining the nucleus that binds chromatin and contributes to spatial genome organization; however, due to the many other functions of lamins, studies knocking out or altering the lamin polymer cannot clearly distinguish between direct and indirect effects. To overcome this obstacle, we specifically targeted the mapped histone-binding site of A/C lamins by microinjecting antibodies specific to this region predicting that this would make the genome more mobile. No increase in chromatin mobility was observed; however, interestingly, injected cells failed to go through mitosis, while control antibody-injected cells did. This effect was not due to crosslinking of the lamin polymer, as Fab fragments also blocked mitosis. The lack of genome mobility suggested other lamin-chromatin interactions. To determine what these might be, mini-lamin A constructs were expressed with or without the histone-binding site that assembled into independent intranuclear structures. HP1, CenpB and PML proteins accumulated at these structures for both constructs, indicating that other sites supporting chromatin interactions exist on lamin A. Together, these results indicate that lamin A-chromatin interactions are highly redundant and more diverse than generally acknowledged and highlight the importance of trying to experimentally separate their individual functions.

## 1. Introduction

Lamins are important contributors to gene regulation and spatial genome organization. Cells from patients with the lamin A-linked Hutchison-Gilford progeria syndrome (HGPS), as well as certain cardiomyopathy lamin A mutations exhibit aberrant nuclear positioning of certain chromosomes [1,2,3]. Similarly, partial disruption of lamin B1 resulted in the mislocalization of two chromosomes in mouse cells [4]. Altered heterochromatin distribution was additionally observed in HGPS patient cells [5,6], and strikingly, lamin A and lamin B receptor (LBR) knockouts inverted the normal peripheral organization of heterochromatin in photoreceptor cells from nocturnal mammals [7,8]. These very exciting functions of lamins are slightly tempered by the fact that lamins have also been implicated in splicing [9], DNA replication [10,11,12,13,14], transcriptional regulation by transcription factor binding [15], cell cycle regulation [16,17], ROS signaling and senescence [18], mechanotransduction [19,20], cell mechanical stability [21,22,23,24] and cell migration [24,25]. Therefore, it is difficult to distinguish specific effects of disrupting lamin-chromatin interactions from secondary or tertiary effects due to the disruption of the many lamin functions in experiments using knockouts or disease alleles. 

Lamins are reported to bind directly to DNA [26,27,28], to core histones [29,30,31] and beta-heterochromatin [32], as well as hundreds of other proteins, including transcription factors [33,34]. Thus, even lamin-genome interactions are complex and difficult to decipher. Lamins have a short N-terminal “head” domain followed by a coiled-coil “rod” domain of ~52 nm and end in a C-terminal "tail" domain that is thought to be mostly unstructured with an Ig-fold in the middle ~1/3 of the tail. These characteristics are shared by the four predominant lamin subtypes, A and C, encoded by the *LMNA* gene and B1 and B2, encoded respectively by the *LMNB1* and *LMNB2* genes. The first mapped chromatin-binding site on lamins was in the rod [35], and subsequently, the reported DNA binding to matrix-associated regions (MARs) was found to reside in this region [36]. At the same time, the finding that the rod of the cytoplasmic intermediate filament vimentin also bound DNA suggested that the rod interaction might be a non-specific interaction based on general properties of intermediate filament coiled coils [28]. A specific high-affinity binding site for core histones (~300 nM) was mapped to the beginning of the tail domain (residues 396–430) using a series of human lamin C (a shorter splice variant of lamin A) truncation mutants [31]. This site was in a region shared by both lamin A and lamin C. A later study on *Drosophila* lamin Dm0 (a B-type lamin) found that specific histones H2A/H2B bind this lamin and determined that there were two chromatin-binding sites in the *Drosophila* lamin B tail, the first partially overlapping with the mapped region for A/C lamins (residues 425–473) in the beginning of the tail and the second towards the end of the tail (residues 572–622) [29].

To specifically target the principal mapped histone-binding site of A/C lamins, we used antibodies generated to a peptide encompassing the mapped site [37]. These were microinjected, and cells stably expressing GFP-labelled chromatin regions were assayed for changes in chromatin mobility, finding no increased mobility. Interestingly, however, it was observed that cells microinjected with the histone-binding site antibodies failed to enter mitosis, potentially revealing an unexpected function for lamin-chromatin binding. Separately, we expressed a “mini-lamin” lacking 4/5 of the rod (A∆rod) that assembled internal nuclear structures similar to those reported for several lamin A point mutations associated with human disease [38,39,40]. Only certain types of chromatin or chromatin proteins accumulated around the lamin A∆rod structures, including promyelocytic leukaemia protein (PML), centromeric protein CenpB, heterochromatin protein HP1 and the silencing mark it binds H3K9me3, but not the peripheral silencing histone mark H3K9me2, DNA damage protein 53BP1 or γH2AX. Surprisingly, these chromatin proteins also interacted with structures formed by the control in which the mapped histone-binding site is additionally deleted, indicating that another region on lamin A can directly or indirectly bind these specific chromatin types.

## 2. Materials and Methods

### 2.1. Plasmid Construction

The human lamin A coding sequence was amplified by PCR with primers that added 5′ Bam HI/Nde 1 and 3′ Not 1 sites. To produce A∆rod, these primers were used with internal primers containing Hind III sites that fused nucleotides 203 and 1012 via an added alanine codon (sequence AGCTT; amino acid 68 fused to 338). To generate the A∆rod∆hbs mutant, the A∆rod construct was further deleted for the known histone-binding site (amino acids 396–429; nucleotides 1185–1287) [31] by using internal primers with a SpeI site replacing nucleotides 1178–1184 and upstream of nucleotide 1288. These genes were moved to the cytomegalovirus (CMV)-driven pHHS10B HA epitope tagged vector for mammalian transfection.

### 2.2. Cell Culture and Transfections

All cells including both unmodified and modified U2OS, HeLa, COS-7 and HT1080 cell lines were maintained in high glucose DMEM supplemented with 10% foetal bovine serum (FBS), 100 µg/mL penicillin and 100 µg/mL streptomycin sulphate. The CenpB-GFP stable U2OS line was obtained from Kevin Sullivan [41] and the H2B-GFP stable HeLa line from Geoff Wahl [42]. Both lines were maintained under selection with G418 at 500 µg/mL. LacO integrated HT1080 cell lines were obtained from Wendy Bickmore [43]. Line B49.5.1 contains an integration into chromosome 5 in a euchromatic region, and line B49.2.7 contains an integration into chromosome 13 in a heterochromatic region. Selection for the LacI-GFP in the LacO lines was maintained with 100 µM hygromycin and 5 µM blasticidin S HCL. DNA was transfected 12 h after plating using Fugene 6 (Roche) according to the manufacturer’s instructions.

### 2.3. Antibodies

Antibodies against the lamin A/C histone-binding site were previously described (rabbit polyclonal 3262; [37]) and were generated to residues 396–429 (QRSRGRASSHSSQTQGGGSVTKKRKLESTESRSS) based on the mapped chromatin binding site from residues 396–430 [31]. Lamin B2 antibody was a guinea pig antibody generated to the sequence at the same relative position in human lamin B2 [37]. Other antibodies include pan-lamin antibody 8188 (McGowan lab, Scripps Research Institute, La Jolla, CA, USA), HA-tag rabbit polyclonal Y11 (Sc-805, Santa Cruz Biotechnology, Dallas, TX, USA) and HA-tag mouse monoclonal (Covance, Princeton, NJ, USA), CenpC rabbit polyclonal 554 (Earnshaw lab, University of Edinburgh, Edinburgh, UK), H3K9me2 rabbit polyclonal (pAb 060-050, Diagenode, Denville, NJ, USA), H3K9me3 rabbit polyclonal (056-050, Diagenode, Denville, NJ, USA), HP1 (MAB3584, Merck Millipore, Billerica, MA, USA), 53BP1 (NB100-304, Novus Biologicals, Littleton, CO, USA), γH2AX (05636, Merck Millipore, Billerica, MA, USA), Rb mAb 4H1 (9309, Cell Signaling Technology, Danvers, MA, USA), phospho-Rb S780 (9307S, Cell Signaling), BrdU (mAb B44, Becton Dickinson, Oxford, UK), histone H2A (mAb #3636, Cell Signaling Technology, Danvers, MA, USA) and nucleolin (ab22758, Abcam, Cambridge, UK). PML, CenpA and CenpB mouse monoclonal antibodies were kind gifts from Kevin Sullivan (University of Galway). To stain for ER/nuclear membrane DiOC_6_ (Thermo Fisher Scientific, Waltham, MA, USA) was used. Secondary antibodies were all donkey minimal cross-reactivity reagents from Jackson ImmunoResearch Laboratories, West Grove, PA, USA.

Antibodies were affinity purified against the protein fragment/peptide used in their generation. The antibody baits were dialyzed out of their storage buffer into PBS and coupled to Affi-Gel matrix. Antibodies were bound to the column from serum, eluted with 200 mM glycine pH 2.3, and the buffer was immediately exchanged using spin concentrators to PBS containing 25% glycerol. For microinjection affinity purified antibodies were coupled using sulfo-SMCC (sulfosuccinimidyl 4-(*N*-maleimidomethyl)cyclohexane-1-carboxylate) crosslinker to a peptide of the nuclear localization signal (NLS) of the SV40 T antigen and stored frozen in glycerol.

Fab fragments were generated by papain cleavage using a papain agarose slurry. Briefly, antibody was concentrated to a 750-µL volume at 12 mg/mL, then dialyzed against papain agarose slurry equilibration buffer (25 mM NaOAc, 20 mM cysteine, 10 mM EDTA, pH 6.2) in a Millipore spin filter (UF 10 K). Concentrated antibody was used to re-suspend a 750-µL volume of pre-equilibrated papain agarose beads (washed 5×) and incubated for 4 h at RT with rotation. The mixture was incubated for a further 45 min at 37 °C and spun down at 1500 rcf to remove beads, before the addition of 0.1 volumes of 1 M Tris pH 8.0 to the supernatant. Supernatant was loaded onto a protein A column pre-equilibrated with 100 mM Tris pH 8.0. Fab fragments were eluted from the column using 100 mM Tris pH 8.0, followed by 10 mM Tris pH 8.0. Eluted Fab fragments were dialyzed and concentrated using PBS in 10 K molecular weight cut-off spin concentrators.

### 2.4. Testing Antibody Blocking

Lamin A∆rod protein fused to a 6x-histidine tag was expressed in BL21-(DE3) cells by induction with 0.3 mM isopropyl-1-thio-β-d-galactopyranoside at A595 0.7 for 3 h at 37 °C, collected by centrifugation and lysed by sonication in 25 mM HEPES, pH 8.0, 0.1 mM MgCl_2_, 3 mM β-mercaptoethanol containing 1 mM phenylmethylsulfonyl fluoride, 1 µg/mL aprotinin, 1 µM leupeptin and 1 µM pepstatin. The protein was isolated from inclusion bodies after pelleting with a 20-min centrifugation at 27,000× *g*, then washing with 1% Triton X-100 and resuspending in 20 mM HEPES, pH 8.0, 8 M urea, 3 mM β-mercaptoethanol. Further purification was achieved by incubation with nickel resin (Qiagen, Germantown, MD, USA), washing, eluting with the same buffer containing 200 mM imidazole and finally dialysis into 20 mM Tris-HCl, pH 8.0, 8 M urea, 2 mM dithiothreitol, 1 mM EDTA with protease inhibitors. 

For histone purification, a HeLa cell pellet was resuspended in 0.1 M H_2_SO_4_ and incubated at 4 °C with rotation for 2 h. The lysate was spun to pellet cell debris, and an equal volume of 1 M Tris-Cl, pH 8 was added to neutralize the supernatant along with NaCl to 0.5 M, EDTA to 2 mM and DTT to 1 mM final concentration. This was passed through SP Sepharose fast flow (Sigma-Aldrich, St. Louis, MO, USA) pre-equilibrated with 50 mM Tris-Cl pH 8.0, 0.5 M NaCl, 2 mM EDTA, washed with the same buffer with salt increased to 0.6 M NaCl and histones eluted in the same buffer with 2 M NaCl. Histones were precipitated by overnight incubation with 4% perchloric acid at 4 °C, then washed in 4% perchloric acid acidified acetone and acetone. After this, they were resuspended in _dd_H_2_O, 0.5 mM PMSF. 

The lamin A∆rod protein was covalently coupled to Affi-gel 10 (Bio-Rad, Hercules, CA, USA) at room temperature for 1 h, as this could be accomplished in urea buffer (50 mM HEPES, 6 M urea, 1 mM EDTA, 1 mM DTT, 1 mM PMSF in this case) to maintain bound lamins as individual molecules or dimers. Degree of coupling was measured by lamin depletion in the flow-through and constituted 0.7 mg per 1 mL of beads. The lamin A∆rod beads were then exchanged with PBS containing 1 mM PMSF, divided into three parts and incubated for 1 h at room temperature with: (1) 4 volumes of the same buffer; (2) 4 volumes of the same buffer containing 10× molar excess of affinity purified antibodies to BSA; or (3) affinity purified antibodies to the lamin A/C histone-binding site. After washing with 20 volumes of PBS, the buffer was exchanged again to histone buffer (50 mM Tris-HCL pH 7.4, 70 mM NaCl, 1 mM DTT, 1 mM PMSF), and core histones were added in 4× molar excess and allowed to incubate with rotation for 1 h at room temperature. The beads were then sequentially washed with 30 volumes of histone buffer and eluted with 1 volume of the same buffer containing 0.5% SDS, separated by SDS-PAGE and processed by standard procedures for Western blot using histone H2A antibodies (Cell Signaling, Mouse mAb #3636) and a LiCor Odyssey for quantification.

### 2.5. Microinjection

CenpB-GFP U2OS and H2B-GFP HeLa cells were plated onto 25-mm coverslips in 35-mm dishes and synchronized with a double thymidine block using 200 µg/mL thymidine and washing after 15 h with 3 PBS and 3 media exchanges over the course of 1 h after the first block. After 12 h, the second thymidine treatment was applied, and 15 h later, the thymidine was removed with 6 exchanges of PBS/media over 30 min. Within a week prior to an injection experiment, an NLS-conjugated antibody aliquot was dialyzed to remove glycerol and concentrated to ~10 mg/mL in PBS. This was then mixed with Texas red-labelled BSA (Jackson ImmunoResearch Laboratories, West Grove, PA, USA) that lacked an NLS or FITC-dextran (150 kD; Molecular Probes). Cells were then injected in the nucleus using an Eppendorf microinjector in manual mode. At the specified times after injection, cells were either moved to chambers for live cell imaging or fixed and processed for immunofluorescence microscopy.

For B49.5.1 and B49.2.7 cell lines, cells were plated at ~40% confluency and synchronized with a double thymidine block using 2 mM thymidine and washing after 12 h with 3 PBS and 3 media exchanges. After a 12-h release, the second 2 mM thymidine treatment was applied, and 12 h later, thymidine was removed by 3 PBS washes. At this point, cells were trypsinised and plated in 35-mm dishes with 25-mm cover glass bottoms at ~25% confluency. At 12 h post thymidine release, cell media were supplemented with 25 mM HEPES pH 7.4, and cell nuclei were microinjected with a mixture of NLS-conjugated lamin A/C histone-binding site affinity purified IgG and Dextran-Texas Red (70 kD; Molecular Probes) in PBS. Cells were injected using a FemtoJet microinjector (Eppendorf, Hamburg, Germany) and InjectMan micromanipulator (Eppendorf) and microinjection needles pulled on a P-97 micropipette puller (Sutter Instrument, Novato, CA, USA) from borosilicate glass GC120F-10 (Warner Instruments, Hamden, CT, USA). 

### 2.6. Microscopy

For live cell microscopy of the CenpB-GFP and H2B-GFP lines, coverslips were moved to a closed Dvorak-Stottler chamber with fresh CO_2_-exposed media supplemented with 25 mM HEPES pH 7.4. Images were obtained using a Zeiss Axiovert S100TV microscope interfaced to a Bio-Rad MRC1024 confocal system with 488-nm, 568-nm and 647-nm krypton/argon laser lines with the stage heated by a constant heated airflow. Images were taken using 10% laser power and at the frequencies noted, all of which were initially tested on each cell line for the frequencies and durations used for long-term viability. For LacO-integrated HT1080 cell lines, after microinjection, cells were imaged using a Zeiss 880 laser-scanning confocal microscope equipped with an XL multi S1 incubator and CO_2_ humidity chamber to maintain a constant temperature of 37 °C and 5% CO_2_ level. Z-stacks were collected (0.6 µm steps) sampling multiple cells in different regions of the plate every 10 min for 3 h.

Fixed cell images were taken on either a Nikon TE-2000 microscope equipped with a 1.45 NA 100× objective, a Sedat quad filter set, a PIFOC Z-axis focus drive (Physik Instrumente, Karlsruhe, Germany) and a CoolSnapHQ High Speed Monochrome CCD camera (Photometrics, Tucson, AZ, USA) run by Metamorph image acquisition software, or a DeltaVision system (Applied Precision, Inc., Mississauga, ON, Canada), or a Zeiss 880 Airyscan confocal system. Image stacks (0.2-µm steps) were deconvolved from the Nikon microscope using AutoquantX and from the DeltaVision using SoftWoRx Version 2.5 or 3.0. Airyscan images were processed by using Zeiss Zen software and Airyscan processing. Micrographs were saved from source programs as 12-bit .tif files and analysed with Image Pro Plus software and/or prepared for figures using Photoshop 8.0. Images were loaded on OMERO (www.openmicroscopy.org) and adjusted for display [44].

### 2.7. Image and Bioinformatics Analysis and Statistics

The position of CenpB-GFP centromere spots was followed using Imaris 8 software (Bitplane, Zürich, Switzerland). Spot position was recorded every minute for 10 min, and an average movement speed was calculated. Additionally, movement amplitude was estimated for each centromere as the maximal distance between CenpB spots on the same track. Kolmogorov–Smirnov tests were performed to compare injected and non-injected datasets. The position of LacO arrays was assessed using ImageJ to create max intensity Z projections, then measuring the distance from the LacO-LacI GFP spot, visible as a small region of max pixel intensity, to the edge of the nucleus delimited by both the diffuse LacI-GFP signal and the co-injected fluorescent dextran. Imaris 8 software (Bitplane) was used to create 3D reconstructions from deconvolved serial z-sections.

## 3. Results

### 3.1. Microinjection of Lamin Histone-Binding Site Antibodies Has Little Effect on Overall Genome Mobility

**Figure 1:**
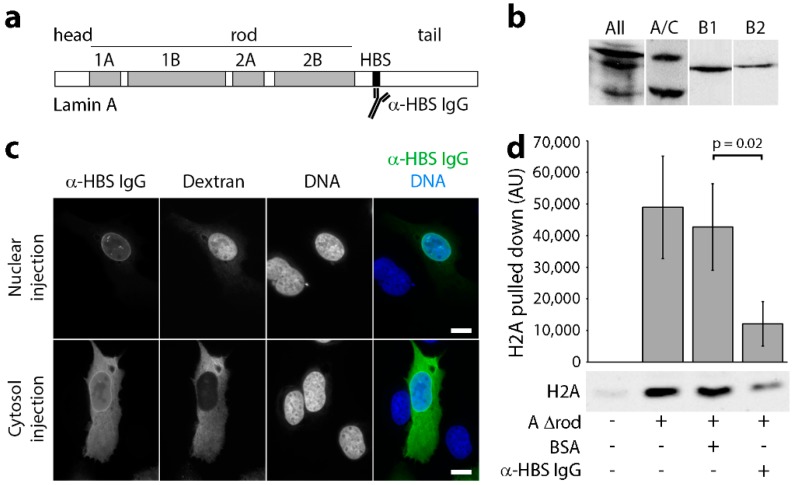
Lamin A/C histone binding site antibodies are specific, target in vivo and block H2A binding in vitro. (**a**) Schematic of lamin A domain structure highlighting the head, rod and tail domains, as well as the mapped chromatin binding site (HBS; Taniura et al., 1995). (**b**) Western blot on total HeLa cell lysates using a pan-lamin antibody and each histone-binding site antibody (A/C, B1 and B2) revealed that all antibodies prepared against the chromatin binding sites of lamins were each highly specific for each subtype. (**c**) The affinity-purified antibodies were conjugated to an SV40 NLS peptide and microinjected into either the nuclei or cytoplasm of U2OS cells. In both cases, subsequent fixation and visualization with fluorophore-conjugated secondary antibodies revealed nuclear rim staining, consistent with their binding the expected target on lamin A in the polymer underlying the inner nuclear membrane. (**d**) To test for antibody blocking of histone binding, lamin A∆rod protein was coupled to beads, incubated with either no antibodies (Lane 2), antibodies generated against BSA (Lane 3) or the lamin A histone-binding site antibodies (Lane 4). Uncoupled beads were also tested as a control for background binding to the beads because histones tend to be sticky (Lane 1). After washing, each was incubated with histones, eluted with SDS and analysed for the amount of bound histones by Western blot. Three technical replicates were quantified for fluorescence intensity using a LiCor Odyssey and plotted (n = 3). Standard deviations are shown, paired t-test shows a significant reduction in H2A bound by lamin A∆rod in the presence of the histone-binding site antibody compared to the BSA control. Scale bars, 10 µm.

To be able to specifically target the mapped histone-binding site of endogenous lamins A/C (Figure 1a, residues 396–430; [31]) without disrupting the endogenous polymer, we microinjected antibodies generated against this region. A polyclonal antibody was previously generated to a peptide encompassing this region (residues 396–429; [37]), and both these and similarly generated antibodies to the other lamin subtypes were highly specific for each subtype (Figure 1b). The antibodies were affinity purified against the peptide used to generate them, concentrated to ~10 mg/mL and microinjected into the nuclei of U2OS cells stably expressing the centromeric protein CenpB fused to GFP. The hypothesis was that antibody binding to lamins would disrupt lamin-chromatin interactions, and consequently, this would increase the mobility of the genome due to a loss of physical tethering to the relatively stable lamin polymer. As expected, the microinjected antibodies targeted the lamin polymer underlying the nuclear membrane, yielding the canonical rim staining when subsequently fixed and visualized with secondary antibodies (Figure 1c). We also tested whether the antibodies could really block histone binding to lamins by coupling lamins to a support matrix while in urea, so that they bound as individual molecules or dimers, incubating these in batch with either control antibodies or with the lamin A/C histone-binding site antibodies, washing these and then incubating with a fraction of core histones isolated from HeLa cells. After washing, the addition of SDS released bound proteins that were run on Western blot and quantified. The data, after subtracting background histone binding to the matrix, revealed a 71.5% reduction in histone binding for the sample pre-treated with the lamin A/C histone-binding site antibodies (Figure 1d). It should be noted that while this was not a complete block, the concentrations used in vitro were necessarily orders of magnitude lower than the 10-mg/mL concentration of antibodies injected into cells. 

**Figure 2:**
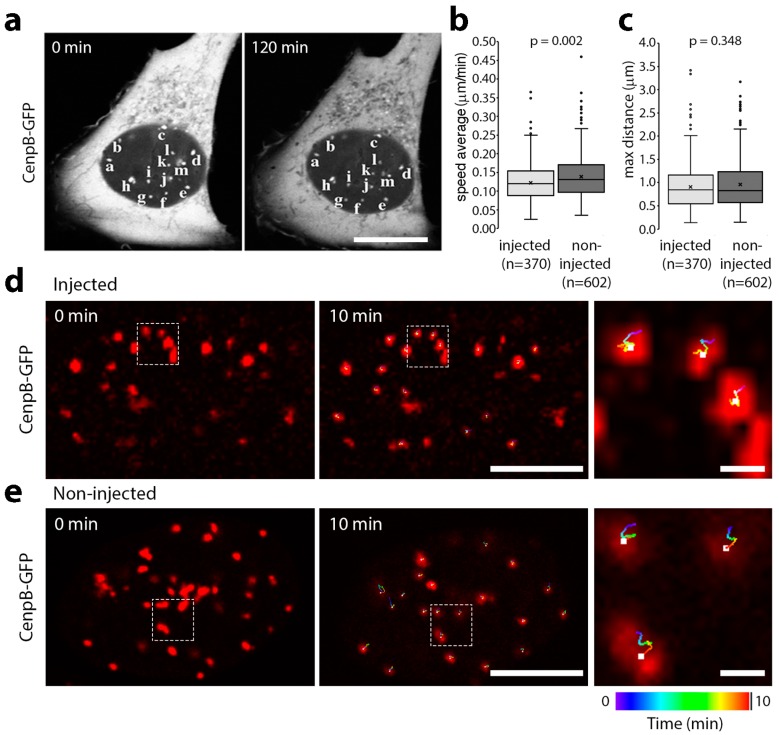
Microinjection of lamin A/C chromatin binding site antibodies does not increase the mobility of centromeres. (**a**) The affinity-purified antibodies were conjugated to an SV40 NLS peptide and microinjected into either the nuclei or cytoplasm of U2OS cell stably expressing CenpB-GFP. With images taken every 10 min the positions of centromeres were essentially unchanged after 2 h. (**b**) Movies covering a period of 10 min with images taken every 1 min were analysed using Imaris 8 tracking software. The data for mobility were plotted using a Tukey box-plot with the median represented by a line and the mean by an x. The *p*-value from a Kolmogorov–Smirnov test is given above. (**c**) The same movies were also analysed after tracking to determine the two most distal points in the area covered by the centre of the CenpB-GFP signal. The data for distance were plotted using a Tukey box-plot with the median represented by a line and the mean by an x. The *p*-value from a Kolmogorov–Smirnov test is given above. No significant differences were observed between histone-binding site antibody injected and non-injected cells. (**d**) Tracking of the movement of the CenpB-GFP centromere spots was followed for each frame in the 10-min movies. The position of centromeres at the start of the movie is shown in the first panel, while the middle panel shows the final position with the tracking for the centre of the GFP signal generated using Imaris 8 software. Centromeres were false coloured in red to make it easier to visualize the tracking, which is colour-coded for time from blue to green to yellow and then to red and terminating at the white dot at the end of the movies (see the colour bar at the bottom of the figure). In the far right panel, an enlargement from the middle image is shown. (**e**) The same is shown for a non-injected cell. Scale bars, 10 µm.

For longer movies of several hours, images were taken every 10 min to avoid photodamage and bleaching over time. Surprisingly, even without adjusting for possible nuclear rotation, the positions of centromeres were almost identical in most injected cells even after 2 h (Figure 2a). It is possible that chromosome territories restrict more global repositioning; so to determine if more local mobility was increased, shorter movies were generated with images taken every 1 min. These were analysed using Imaris tracking software revealing a median speed in lamin A/C histone-binding site antibody injected cells of 0.12 µm/min travelled per centromere and of 0.13 µm/min in adjacent non-injected cells (Figure 2b). A Kolmogorov–Smirnov test found this to be significant, although it is unlikely that a <10% change in mobility would be biologically relevant. There was also very little difference in the range of movement over time with the greatest distance between any two points within the area sampled by the middle of a centromere spot in the microinjected cells 0.85 µm and in adjacent non-injected cells 0.83 µm (Figure 2c). For both metrics, there was a similarly wide range of individual movements among centromeres within and between cells as indicated by traces showing the full range of movement over the course of movies for individual injected (Figure 2d) and non-injected (Figure 2e) cells.

**Figure 3:**
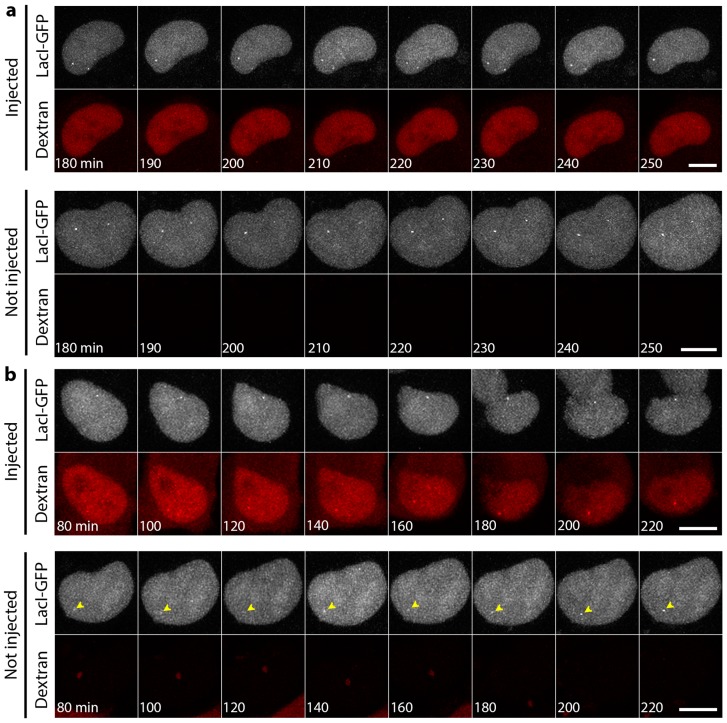
The lack of a mobility effect upon microinjection of lamin A/C histone-binding site antibodies is observed for both euchromatic and heterochromatic regions. (**a**) An HT1080 cell line with a 254 copy LacO array integrated into a euchromatic region of chromosome 5 that tended to be in the nuclear interior was microinjected with lamin A/C histone-binding site antibodies. At 3 h post-injection, the cell was imaged live for 70 min with Z-stacks taken every 10 min. For comparison, a non-injected cell in the same field is shown in the bottom panels. No visual increase in mobility was observed in this or any of several other injected cells observed. (**b**) A similar array integrated into a heterochromatic region of HT1080 chromosome 13 that tended to be at the nuclear periphery was similarly analysed, except that imaging was started at 1 h post-injection and continued for 3 h. Arrowheads indicate the LacO array in the bottom movie, as the spot is more difficult to distinguish in this movie. Again, no difference was observed between the injected and non-injected cells, and very little mobility was observed at all. Scale bars, 10 µm.

We considered the possibility that movement might be a function of nuclear position or the epigenetic environment around a particular genome region. Therefore, the experiments were repeated with cell lines carrying LacO array integrations and stably expressing LacI-GFP. One line had the array integrated into a euchromatic region of chromosome 5, while the other had the array integrated into a heterochromatic region of chromosome 13 [43]. The array in the chromosome 5 line tended to reside in the nuclear interior, while the chromosome 13 array tended to reside towards the nuclear periphery [43,45]. Starting at 1 or 3 h post-injection, images were taken every 10 min over a course of 70 min to several hours. No difference in mobility was observed between the lamin A/C histone-binding site antibody injected and non-injected cells for either the euchromatic chromosome 5 (Figure 3a) or heterochromatic chromosome 13 (Figure 3b) lines.

### 3.2. Microinjection of Lamin Histone-Binding Site Antibodies Blocks Mitotic Entry

**Figure 4:**
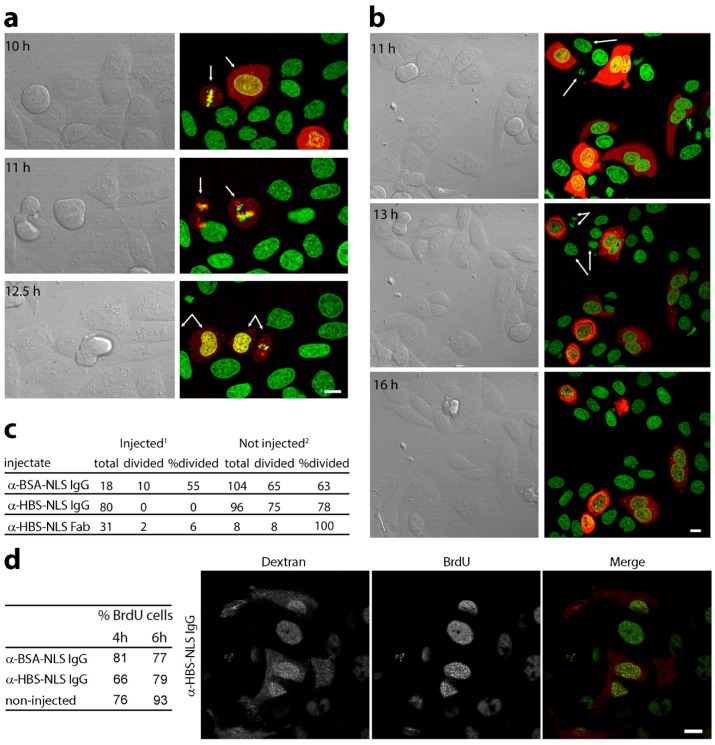
Microinjected lamin A/C HBS antibodies blocked mitotic entry and delayed DNA replication. (**a**–**c**) Synchronized cells were injected within 1 h from release at G1/S with HBS or control antibodies conjugated to an SV40 NLS peptide using fluorescent dextran to identify injected cells. (**a**) Control antibody injected cells (αBSA) visualized from 6 h post-G1/S release (5 h post-injection). Images at times shown follow cells through mitosis with arrows. The control-injected cells went through mitosis at similar rates as non-injected cells in the same fields (**c**). (**b**) Lamin A/C HBS antibody-injected cells were followed longer as no cells entered mitosis in the timeframe of controls. (**c**) Numbers of cells followed for each condition, including also cells injected with Fab fragments of the lamin A/C HBS antibodies. Scale bars, 10 µm. (**d**) HBS antibody effects on DNA replication. HeLa nuclei were microinjected within 30 min after G1/S release and pulsed with BrdU starting at 3 h post-release for 1 h. The percentage of BrdU-positive cells at 4 and 6 h post-G1/S release for each condition is given in the table. To ensure that microinjection itself did not affect DNA replication, control antibodies (BSA-NLS) were injected into a parallel culture. A representative image of an injected, BrdU-labelled cell is shown.

In longer term movies of cells injected with the lamin A/C histone-binding site antibodies, it was noted that cells injected with control antibodies against BSA often were observed to go through mitosis, whereas this was not the case for cells injected with the histone-binding site antibodies. If this was not just an issue of random sampling, it would be a significant finding; so a controlled set of experiments was undertaken to test if microinjection of the histone-binding site antibodies blocked entry into mitosis. Synchronized cells were used so that the cells could be followed visually during the period where they would be expected to enter mitosis, and HeLa cells were used because of their regular and quick cell cycle and reliability to have a reasonable mitotic population even when unsynchronized. In this case, HeLa cells stably expressing H2B-GFP [42] were used to help distinguish the cell cycle stage. The cells were synchronized with a double thymidine block to release them before S-phase and injected within 1 h from thymidine release. As a control, a split of the same cells equivalently treated for the thymidine block was microinjected with similarly affinity purified, NLS-conjugated and concentrated antibodies directed against BSA. After 5 h, coverslips were moved to a chamber for live cell imaging, and cells were then followed, taking images every 15 min for an additional 6–9 h. No notable changes in chromatin distribution based on the H2B-GFP signal were observed over the course of experiments for either the control BSA antibody or lamin histone-binding site antibody injected cells. For both BSA antibody microinjected cells and non-injected cells in the same fields, roughly 60% of the populations had gone through mitosis by 12.5 h post-thymidine release (Figure 4a,c). Surprisingly, no cell injected with the lamin A/C histone-binding site antibodies was observed to enter mitosis during the course of an experiment, even when the duration of imaging was extended out to 16 h post-thymidine release (Figure 4b,c). Notably, at this later time, 100% of non-injected cells in the same population had gone through mitosis.

As the lamin polymer disassembles in each mitosis, we considered the possibility that the lamin antibodies were crosslinking lamin molecules and thus preventing disassembly. Therefore, we also produced Fab fragments of the lamin A/C histone-binding site antibodies. The intact IgG molecule has two separate light chains, so separating these as Fab fragments eliminates the possibility of the two separate light chain binding sites crosslinking the lamin molecules while still specifically targeting the mapped histone-binding site. Microinjection of the lamin A/C histone-binding site Fab fragments yielded the same phenotype as the fully-assembled IgG antibodies with only 6% of microinjected cells entering mitosis, while every untransfected cell in the same visual fields went through mitosis in the same14-h period (Figure 4c). 

### 3.3. DNA Replication Is Partly Delayed in Cells Microinjected with Lamin Histone-Binding Site Antibodies

As cells have to initiate S-phase and replicate their DNA before entering mitosis, we considered the possibility that a defect in either of these processes could underlie the failure of cells injected with the lamin A histone-binding site antibodies to enter mitosis. Most late-replicating chromatin is pericentromeric and peripheral heterochromatin, so it is possible that loss of stabilizing connections might yield a defect in DNA replication, and this is reasonably consistent with previous reports of a Lamin B1 role in DNA replication [10,11,12,13,14]. Therefore, the effect of microinjection of the histone-binding site antibodies on DNA replication was tested by BrdU incorporation. 

Cells were microinjected with the lamin A/C histone-binding site antibodies 30 min after release from a thymidine block and pulsed with BrdU for 1 h starting at 3 or 5 h post-thymidine release. Cells were then fixed and stained with anti-BrdU antibodies and the percentage of microinjected cells and non-injected cells quantified. At 4 h post-thymidine release, 66% of cells injected with the antibodies against the lamin A/C histone-binding site were BrdU-positive, while 81% of cells microinjected with the antibodies to BSA conjugated to an NLS were BrdU-positive, and 76% of non-injected cells were BrdU-positive (Figure 4d). At 6 h post-thymidine release, the number of BrdU-positive cells injected with the chromatin-binding site antibodies had largely caught up with the cells injected with control antibodies against bovine serum albumin (BSA; also conjugated to an NLS) (Figure 4d). BrdU staining tended to be diffuse and not indicative of any particular phase of replication (Figure 4d). Therefore, it appears that there is a slight delay in initiating, or increased length of S-phase for cells microinjected with the lamin histone-binding site antibodies at this time, but the cells are not blocked from initiating DNA replication in S-phase.

### 3.4. Mini-Lamin A Structures Accumulate DNA

**Figure 5:**
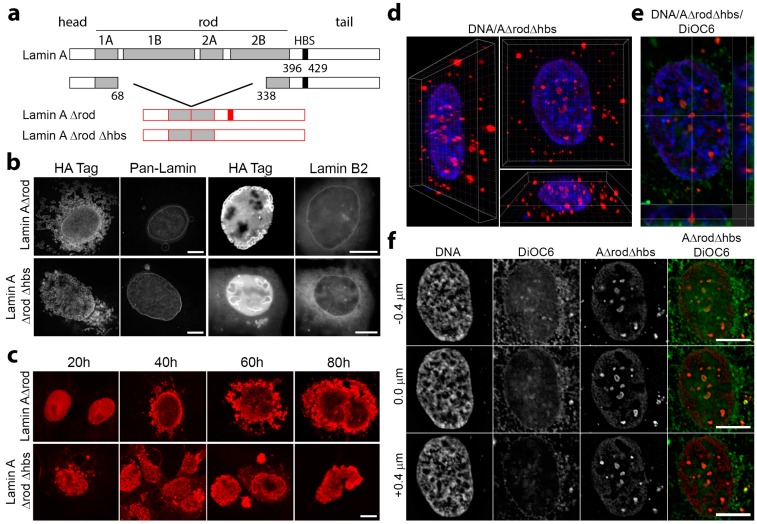
A lamin A mutant generated to increase the density of histone-binding sites assembles intranuclear structures independent of the peripheral lamina. (**a**) The lamin A/C rod domain is similar in mass to the globular tail domain of lamin A that contains the mapped lamin A/C histone-binding site. Thus, in A∆rod, most of the rod was deleted to roughly double the mass density of histone-binding sites. As a negative control, A∆rod∆hbs also deleted the major mapped histone-binding site. (**b**) When A∆rod was expressed in U2OS cells, it formed circular structures both in the nucleus and in the cytoplasm. In most cells, these structures neither integrated with nor perturbed the peripheral lamin structural network, as indicated by co-staining for the lamin A∆rod mutants and for endogenous lamins with a pan-lamin antibody or an antibody to the similar region in lamin B2. (**c**) Expressing the lamin A constructs in COS-7 cells to match the earlier B1∆rod experiments, some A∆rod and A∆rod∆hbs structures were formed by 20 h post-transfection and most by 40 h. (**d**,**e**) 3D reconstructions of cells, produced from serial z-sections (0.2-μm step size) after deconvolution using Imaris 8 software revealed that the A∆ structures tend to be completely internal without connecting to the membrane. (**f**) Co-staining with the membrane dye DiOC_6_ further confirmed that the internal structures were free from membrane, and thus, results could not be attributed to nuclear membrane proteins. All scale bars, 10 µm.

A possible interpretation of the failure to observe increased genome mobility in lamin A/C histone-binding site antibody injected cells would be the presence of other chromatin binding regions on the lamin A protein that maintain stabilizing contacts. At the same time, as lamin A has many binding partners determined by two-hybrid studies [33,34], it is possible that the antibodies have other effects besides the expected interference with histone binding. To get a sense of whether other specific types of chromatin might be affected by blocking the mapped lamin A/C histone-binding site and whether other regions in the lamin A protein might interact directly or indirectly with other specific types of chromatin, we compared mini-lamin A constructs containing the histone-binding site (A∆rod) or deleted for this sequence (A∆rod∆hbs) (Figure 5a). Whereas lamin A knockout released peripheral heterochromatin from the nuclear periphery [8], the prediction was that particular types of chromatin protein might accumulate on the mini-lamin A constructs. Moreover, chromatin proteins that accumulated on both constructs must bind in regions outside the mapped histone-binding site.

The mini-lamin constructs were patterned after a previously characterized B1∆rod mutant in which 4/5 of the rod domain was deleted, yet it still assembled filaments in vitro [37]. It was necessary to retain this 1/5 of the rod to be able to assemble higher order structures because the experiment could not work if the soluble C-terminal domain just diffused throughout the nucleoplasm. The prediction also considered that the mini-lamins might exhibit enhanced recruitment of relevant chromatin proteins because the lamin A/C rod domain is roughly the same mass as the globular tail domain of lamin A that contains the mapped lamin A/C histone-binding site; so deleting the rod would roughly double the mass density of histone-binding sites (Figure 5a). Due to the fact that the rod is linear and measures at ~52 nm, in an assembled polymer, the linear density of histone-binding sites might be expected to be as much as five-fold higher. The A∆rod mutant was expressed in cells fused to an N-terminal HA-tag. Whereas the B1∆rod integrated into the peripheral lamin polymer and promoted massive nuclear lobulation with some lamin subtypes segregated into separate lobules [37], A∆rod assembled into circular structures both in the nucleus and in the cytoplasm with neither integration nor perturbation of the peripheral lamin structural network (Figure 5b). This was confirmed by co-staining with the HA tag of A∆rod and antibodies to endogenous lamins, in one case using a pan-lamin antibody that recognizes a conserved region of the rod domain of all lamin subtypes that is missing in the A∆rod mutants and in the other lamin B2-specific antibody. Interestingly, the lamin B2-specific antibody was generated to the same region at the beginning of the C-terminus where the histone-binding site was identified on lamin C, but has no cross-reactivity with lamin A (Figure 1b). Both lamin antibodies revealed no co-localization with the internal lamin A∆rod and A∆rod∆hbs structures, confirming that endogenous lamins were unaffected by the lamin mutant structures. Interestingly, these circular structures were similar to structures previously observed for several lamin A point mutants linked to human disease [38,39,40]. The A∆rod constructs also differed from the B1∆rod mutant in that it was reported that the B1∆rod phenotype required days to develop [37], while the A∆rod structures could be observed by 20 h post-transfection (Figure 5c). This suggests a need for cells to undergo mitosis for the B1∆rod to integrate into and disrupt the peripheral polymer while for the A∆rod, the lack of overlap between the structures formed and the peripheral lamin polymer argues the opposite.

The structures formed by the A∆ proteins tended to accumulate in the nuclear interior, and so, chromatin distribution changes could not be due to the effects of other nuclear membrane proteins. This was confirmed by 3D reconstructions of the z-series taken through cells (Figure 5d) and by the failure to observe co-localization in the internal nuclear structures with the membrane dye DiOC_6_ that stains both the endoplasmic reticulum and the nuclear membrane (Figure 5e,f).

**Figure 6:**
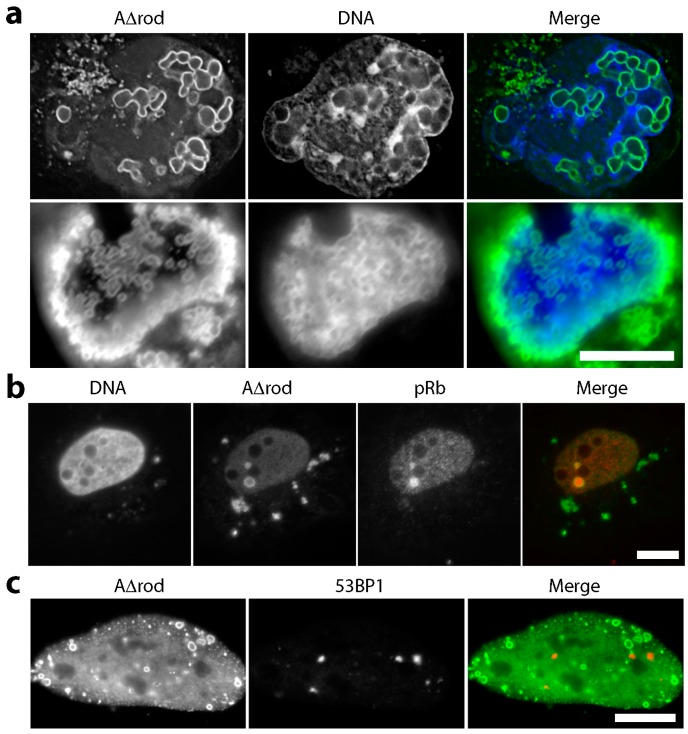

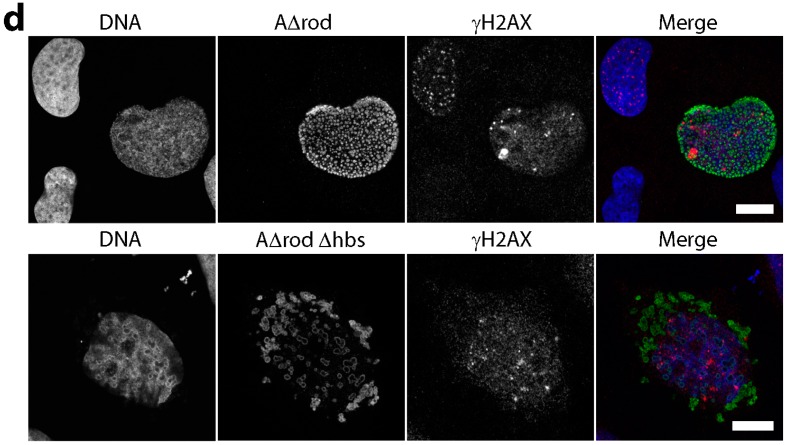
General chromatin effects of A∆rod and A∆rod∆hbs. (**a**) DNA staining (DAPI) in U2OS cells expressing A∆rod. Chromatin staining tended to be stronger on or around lamin A∆rod structures. (**b**) pRb is a known binding partner of lamin A, and so, it was co-stained as a positive control, revealing strong co-localization with the A∆ structures. (**c**) No co-localization between A∆rod structures and 53BP1 in HeLa cells indicates that chromatin is not damaged by the structures. (**d**) Similarly, γH2AX did not accumulate on A∆ structures.

DNA was stained using DAPI in U2OS cells expressing A∆rod. The mutant structures containing a higher concentration of chromatin binding sites exhibited a stronger associated DAPI signal than more distal areas (Figure 6a), suggesting that these structures influence or accumulate heterochromatin. Lamin A is known to bind the retinoblastoma protein pRb in a complex with LAP2α, a soluble splice form of the nuclear membrane protein LAP2β. Therefore, as a positive control, A∆rod cells were stained for pRb expecting to observe a partial co-localization. To our surprise, almost all of the pRb in some cells accumulated on the A∆rod structures, suggesting that, as predicted, the mini-lamin proteins would exhibit denser binding sites for some chromatin partners than the peripheral lamina (Figure 6b). This also highlights the possibility that other soluble proteins in the nucleoplasm could mediate the interactions suggested by the following analysis, and correspondingly, for wild-type lamin A at the nuclear periphery, transmembrane proteins could similarly mediate interactions. As an additional control to check if the mini-lamin proteins yield DNA damage breaks, we also stained for 53BP1 and γH2AX that accumulate at sites of DNA damage, but no overlap in signals was observed (Figure 6c,d). Nonetheless, although many individual untransfected cells exhibited higher numbers of γH2AX foci than the cells expressing A∆rod or A∆rod∆hbs, all A∆rod and 65% of A∆rod∆hbs transfected cells exhibited some γH2AX foci, while only 51% of untransfected cells in the same population exhibited the foci. 

**Figure 7:**
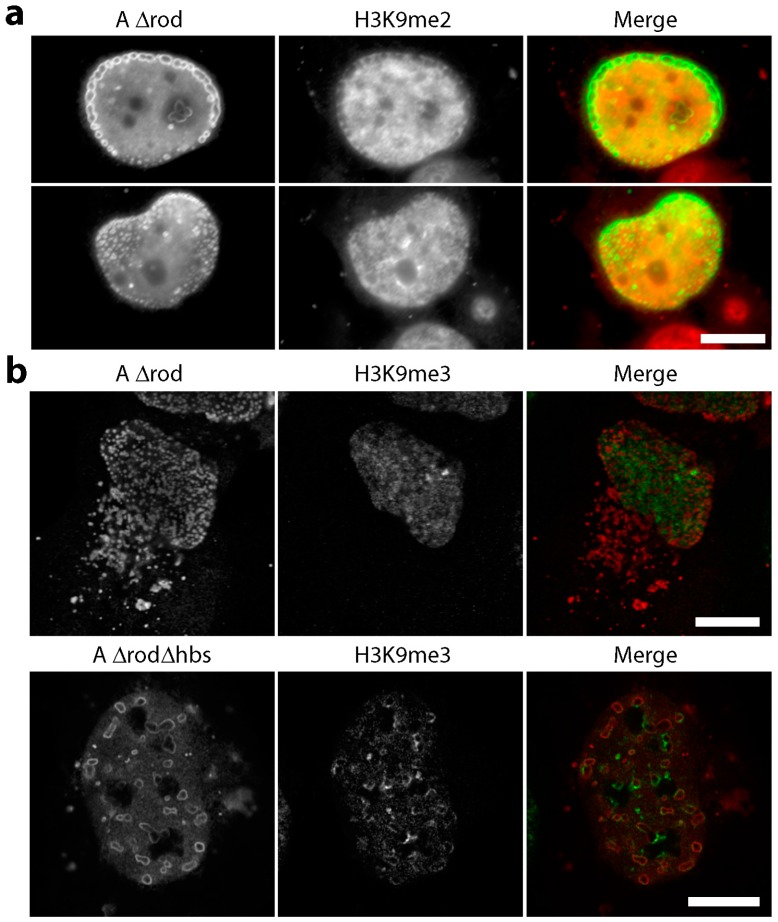

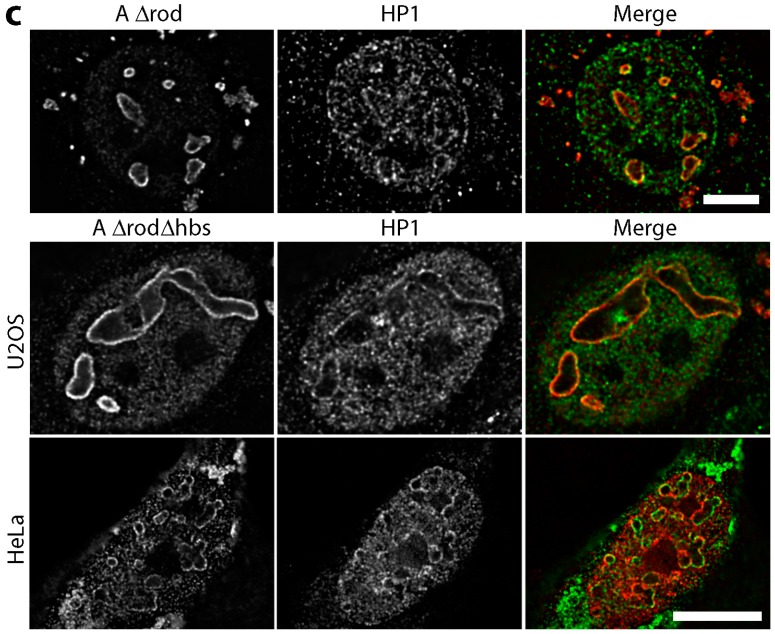
Heterochromatin effects of A∆rod and A∆rod∆hbs. (**a**) H3K9me2 staining in lamin A∆rod expressing U2OS cells. No notable accumulation of these marks was observed on the A∆rod structures. In fact, the greater concentrations of the epigenetic mark tended to occur in regions devoid of the A∆rod structures. (**b**) H3K9me3 staining in lamin A∆rod and A∆rod∆hbs expressing U2OS cells. Clear accumulations of histones carrying this mark were observed around the structures formed by the A∆ proteins. (**c**) HP1 staining revealed concentrations that co-localized with the A∆rod structures in U2OS cells. To test if this was specific to the mapped histone-binding site, cells expressing A∆rod∆hbs were also stained for HP1. The interaction appears to be from a distinct region in the mini-lamins because a similar co-localization was observed. This co-localization with the A∆rod∆hbs structures was observed in both U2OS (upper panels) and HeLa (lower panels) cells. Deconvolved images are shown in this panel to better clarify the co-localization. All scale bars, 10 µm.

For heterochromatin, previous studies have shown that the histone mark H3K9me2 in particular accumulates at the nuclear periphery [46], and there is also a well-known specific interaction between heterochromatin protein 1 (HP1) and the lamin B receptor, a transmembrane protein of the nuclear envelope [47]. HP1 also binds specifically to H3K9me3. Therefore, to specifically check for heterochromatin, cells were separately stained with antibodies to the epigenetic silencing marks H3K9me2, H3K9me3 and to HP1. No notable accumulation of the H3K9me2 marks was observed on the A∆rod structures. In fact, greater concentrations of H3K9me2 tended to occur in areas distal from A∆rod structures (Figure 7a). In contrast, though generally diffuse throughout the nucleoplasm, a specific accumulation of H3K9me3 at or around both the A∆rod and A∆rod∆hbs structures was observed (Figure 7b). As HP1 binds this mark, it is not surprising that HP1 exhibited a similar distribution (Figure 7c, upper panels). To test if this seeming heterochromatin interaction was due to the mapped histone-binding site, the A∆rod∆hbs mutant was also expressed in the U2OS cells, and the same co-localization with A∆rod∆hbs structures was observed (Figure 7c, middle panels). To ensure that this was not a U2OS-specific phenomenon, A∆rod∆hbs was also expressed in HeLa cells and stained with the HP1 antibody, observing the same result (Figure 7c, bottom panels). 

### 3.5. A∆rod Mutants Affect Specific Chromatin Regions Containing Centromere Proteins

**Figure 8:**
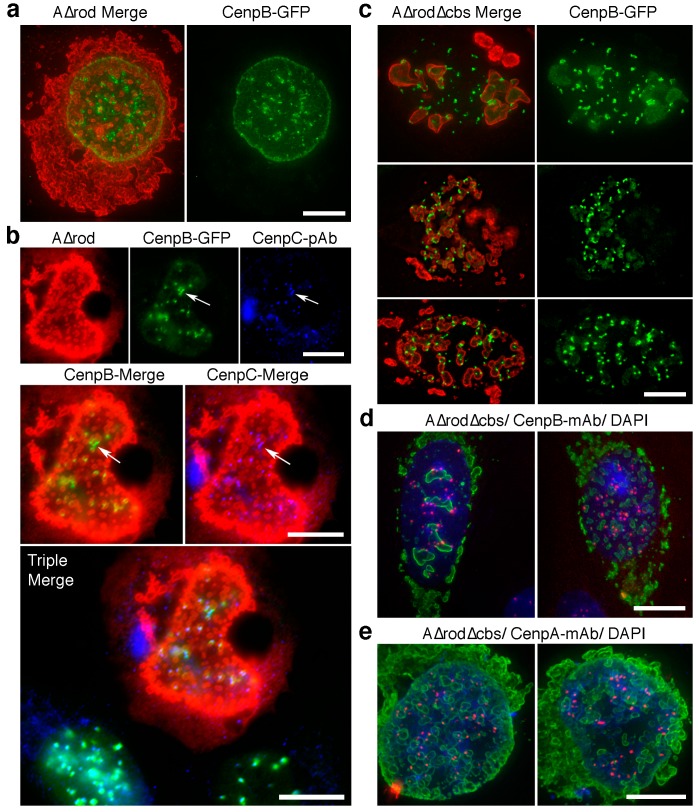
Potential centromere interaction with lamin A. (**a**) U2OS cells stably expressing CenpB-GFP were transfected with A∆rod and imaged. The CenpB-GFP protein accumulated around A∆rod structures both in association with centromere spots and separately, notably at the nuclear periphery. (**b**) To determine if aberrant distributions of the CenpB-GFP also affected endogenous centromere proteins, cells were co-stained with an antibody to CenpC. The CenpC did not display a similar aberrant distribution at the nuclear periphery, but in some cases where CenpB-GFP spots were distended, a similar distension in the CenpC staining pattern was observed. Arrows point to such a distended centromere spot, and both individual and double and triple merged images are shown. (**c**) A similar redistribution of CenpB-GFP is observed in cells expressing A∆rod∆hbs. Notably, the CenpB-GFP signal could typically be observed within the circular structures formed by A∆rod∆hbs. (**d**) To test whether the indicated interaction is an artefact of the exogenously-expressed GFP fusion protein, HeLa cells expressing A∆rod∆hbs and not expressing the GFP protein were stained for endogenous CenpB. Considerable co-localization was observed in roughly half of cells examined, while very little co-localization was observed in the other half. (**e**) HeLa cells expressing A∆rod∆hbs were also stained for CenpA with similar results. All scale bars, 10 µm.

A fortuitous transfection of the A∆rod construct into the CenpB-GFP cells used earlier for antibody microinjection experiments yielded an interesting and unexpected result. Despite there being no observed increase in centromere mobility with the histone-binding site antibody microinjection, the centromere protein used in this experiment appeared to have an affinity for the A∆rod structures. In fact, in cells with many A∆rod circles concentrated around the nuclear periphery, there was an accumulation of CenpB-GFP at the periphery independent of the centromere staining (Figure 8a). To test whether endogenous centromere proteins were also affected by the A∆rod mutant, the CenpB-GFP and HA-A∆rod expressing cells were co-stained with a CenpC antibody (Figure 8b). No accumulation of endogenous CenpC could be observed at the nuclear periphery with the redistributed CenpB-GFP signal; however, the endogenous CenpC was at all intranuclear spots corresponding to CenpB-GFP at centromeres, and in some cases where the CenpB-GFP signal was distorted from a round spot to a more linear configuration on an A∆rod structure, the endogenous CenpC was similarly distorted (Figure 8b, arrow). From this, it can be concluded that the CenpB-GFP interaction with A∆rod is not shared by CenpC, but that the endogenous centromere structure can be distorted by A∆rod interactions with CenpB-GFP.

Just as for the co-localization between HP1 and the lamin A∆ mutants, both the A∆rod and the A∆rod∆hbs had the redistribution effect on the CenpB-GFP (Figure 8c). Commonly, it was observed that the inside of the circular structures formed by A∆rod∆hbs accumulated CenpB-GFP fluorescence (Figure 8c). As excess protein expressed for many GFP fusion proteins accumulates in part in nucleoli, cells were also stained with an antibody to nucleolin, but there was no overlap between the A∆rod∆hbs structures and the nucleolin signal (data not shown). To determine if endogenous CenpB protein also interacts with the A∆rod∆hbs structures, we expressed the A∆rod∆hbs in HeLa cells that did not express the exogenous CenpB-GFP and stained the cells with an antibody to CenpB (Figure 8d). In many cells, a high percentage of CenpB-stained centromeres co-localized with the A∆rod∆hbs structures. For example, in the left panel of Figure 8d, 22 of 39 centromeres visible in the section had partly overlapping pixels with the A∆rod∆hbs structures, even though there were only five such structures visible in the section. However, there were also many cells in which fewer centromeres exhibited co-localization compared to the CenpB-GFP cells, even when many A∆rod∆hbs circles were present throughout the nucleus (e.g., Figure 8d, right panel). For example, in the two CenpB-GFP cells shown in Figure 8c with many A∆rod∆hbs circles throughout the nucleus, only 9.1% of visible centromeres did not exhibit overlapping pixels, whereas in the CenpB-stained cell shown in the right panel of Figure 8d, 26% of visible centromeres did not exhibit overlapping pixels. Of note, no endogenous CenpB signal could be observed at the nuclear periphery or inside the A∆rod∆hbs circles. Thus, there appears to also be an effect on endogenous centromere distribution, suggesting an association with the A∆ mutant structures, but this is facilitated by CenpB overexpression and/or fusion to GFP. HeLa cells expressing A∆rod∆hbs were also stained for CenpA with similar findings (Figure 8e). 

### 3.6. A∆rod Mutants also Affect Pro-Myelocytic Leukaemia Protein Distribution

**Figure 9:**
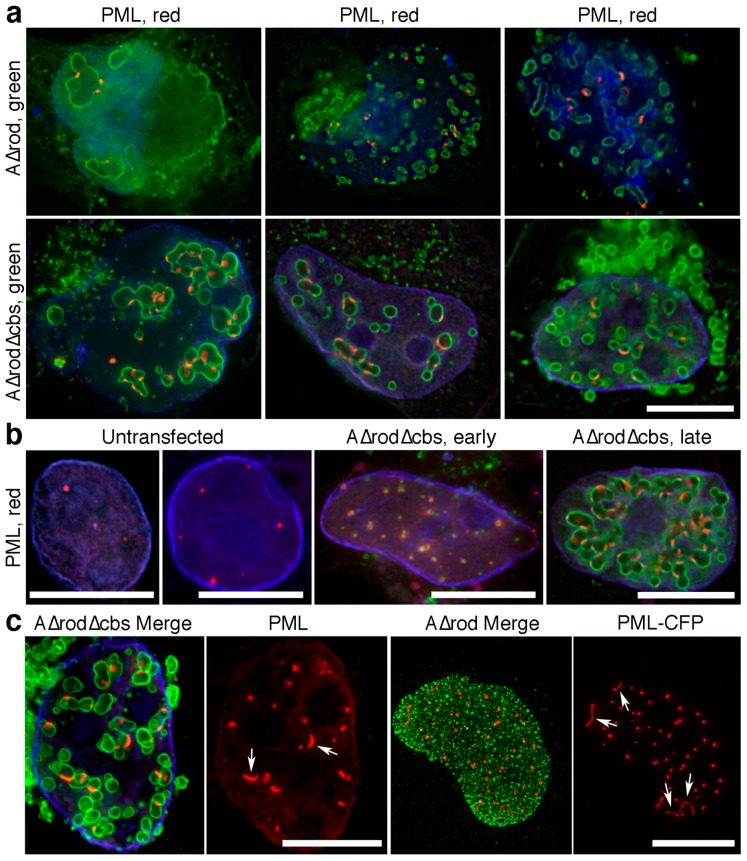
Potential PML interaction with lamin A. (**a**) HeLa cells expressing A∆rod and A∆rod∆hbs were stained with an antibody for PML. Nearly all PML foci were associated with the mini-lamin structures. Scale bar, 10 µm. (**b**) Numbers of observed PML foci increase in cells expressing A∆rod∆hbs. Left panels, untransfected cells exhibited relatively few PML foci. Middle panel, co-localization between A∆rod∆hbs and PML was observed even at early time points before the larger circular structures had formed. Right panel, later cells with fully-formed intranuclear A∆rod∆hbs circular structures exhibited both co-localization with PML and a considerable increase in the number of PML foci. Scale bars, 10 µm. (**c**) PML foci were often distended on the mini-lamin A structures. Left panels show an A∆rod∆hbs expressing cell with two more distended PML foci indicated by arrows that curve around the A∆rod∆hbs circular structures instead of appearing as a normal spot. Right panels show that, in HeLa cells co-expressing A∆rod and PML fused to CFP, extremely long distended PML structures (arrows) were observed, even in cells without significant development of the A∆rod circular structures. Scale bars, 10 µm.

PML is a core constituent of PML/ND10 bodies that are associated with many functions from transcription to cellular stress [48]. Cells expressing A∆rod constructs were stained with an antibody to PML. PML protein accumulated at both A∆rod and A∆rod∆hbs structures (Figure 9a). A typical cell has 8–12 PML foci, but interestingly, cells expressing A∆rod mutants exhibited an increase in the number of PML-stained foci with many A∆rod∆hbs cells having 40–50 PML stained foci (Figure 9b). Notably, even at early times post-transfection before expressed A∆rod∆hbs had assembled into circles and where the staining was very punctate, there was still a co-localization with the PML protein and an increase in the number of PML-stained foci. Similarly to how the CenpB-GFP centromere spots were sometimes observed to be stretched along the surface of an A∆rod structure, it was common to observe a subset of PML foci in a cell being stretched along the surface of an A∆rod or A∆rod∆hbs structure (Figure 9c, left panels). Interestingly, when A∆rod was co-transfected with a PML-CFP construct instead of staining with PML antibodies, the PML-CFP often formed aberrant linear structures that were sometimes associated and sometimes not with the A∆rod spots (Figure 9c, right panels). 

## 4. Discussion

Lamin A knockout or mutation has profound consequences on the orientation of heterochromatin in the nucleus and the spatial positioning of several chromosomes [1,2,3,5,8,49]. However, due to the many structural and other functions of lamin A, it could not be determined with certainty whether these effects were the result of lamin A binding to chromatin, recruitment of genome regulatory proteins or epigenetic enzymes, lamin A indirect recruitment of such proteins through other binding partners, such as nuclear envelope transmembrane proteins that depend on lamin A for targeting, or mechanical weakening of the nucleus. We had predicted that the ability to more specifically target the mapped histone-binding site of lamin A for core histones by antibody microinjection would distinguish these possibilities; however, instead, our results highlight the difficulties of measuring chromatin changes with focused targeting likely because of redundancy in lamin-chromatin and other nuclear membrane-chromatin interactions. 

Although it is not clear whether interactions suggested by the accumulation of HP1, H3K9me3, CenpB and PML are direct or indirect, it is perhaps not surprising that there would be additional chromatin-binding sites on lamin A. A separate study on the *Drosophila* B-type lamin found two separate sites in the C-terminal region [29], and the study mapping this lamin A/C site [31] used lamin C, which is identical to lamin A until 136 amino acids following the end of the mapped histone-binding site and then diverging and truncated using a different exon, while lamin A is 90 amino acids longer than lamin C. Moreover, the mapping study focused on identifying the site responsible for interacting specifically with core histones and used in vitro purified proteins so that this is a very specific and well-characterized interaction [31]. The study finding the histone interactions for *Drosophila* B-type lamin reported that there was no preference for epigenetically-modified histones [29]; however, that study was before many such marks had been identified. We find that the A∆ structures preferentially accumulate H3K9me3 over H3K9me2, and consistent with this, they also exhibit preferential accumulation of HP1. HP1 was previously only reported to interact with the transmembrane protein LBR [47], yet here, we find an accumulation around intranuclear lamin A mutant structures that are separate from the nuclear membrane. Thus, this finding may be an important missing link in explaining the strong effect of lamin A deletion on heterochromatin distributions [7,8]. 

Centromere-nuclear envelope interactions have an interesting history going back over 100 years to the Rabl chromosome configuration, wherein centromeres or telomeres associate with one pole of the nucleus as chromosomes align for meiotic recombination [50]. Early antibody stainings for centromeres revealed similar Rabl conformation in Indian Muntjac cells [51], while work from the Cremer lab revealed that centromere and telomere movements in establishing the Rabl configuration occurred in distinct phases [52]. Additionally, there are stages in the development or growth of certain cell types where centromeres associate with the nuclear periphery. For example, centromeres accumulate at the periphery in G0 and G1 stages for human lymphocytes, and this accumulation is lost as the cells go through the S-phase [53,54]. Notably, in neutrophil differentiation, when major restructuring of the lamina also occurs [55], 100% of centromeres accumulate at the nuclear periphery [56]. In myogenic differentiation, a repositioning of centromeres to the nuclear periphery has also been reported [57], and the importance of centromere positioning in the nucleus is suggested by observations of infertility associated with aberrant positioning [58]. However, there has been no indication of the relevant proteins or mechanism for these centromere redistributions, except in yeast, which do not have lamins where the nuclear envelope transmembrane SUN protein appears to be involved [59] and an indication that a *Drosophila* DNA clone that hybridizes to chromocenters also interacts with *Drosophila* lamins [32]. A role for lamins is at least indirectly indicated by the observation of centromere clustering in cells with the lamin A-linked Hutchison-Guilford progeria syndrome [60] and observations that early embryonic stem cells which lack lamin A have reportedly fewer centromeres at the nuclear periphery [61]. The accumulation of both CenpB and CenpC on the A∆rod structures in the nuclear interior argues that lamin A and soluble nuclear factors are sufficient for these specific and temporal peripheral localizations of centromeres, because nuclear envelope transmembrane proteins, such as the SUN proteins, are restricted to the nuclear periphery. Though further work will clearly be needed to fully understand the mechanism, this finding provides an important first step towards identifying the other factors involved. 

Less has been reported on PML associations with the nuclear periphery. PML protein is normally associated with PML/ND10 bodies that co-stain with proteins, such as Daxx, Sumo and SP100, but when cells undergo nuclear rupture and nuclear contents are released into the cytoplasm, a pool of PML is retained in the cytoplasm that does not interact with these normal partners [62]. The finding of PML protein on the A∆rod and A∆rod∆hbs structures could either indicate a completely novel PML-specific function with lamin A, a novel PML/ND10 body function with lamin A or an aspect of the molecular mechanism of the centromere interaction. The centromere protein CenpC has been reported to interact with Daxx [63] that in turn associates in PML/ND10 bodies [64]. Similar to how centromeres can have cell cycle-specific interactions with the nuclear envelope, it has also been suggested that they can have cell cycle-specific interactions with PML/ND10 bodies, at least in the case of CenpC during viral infection [65]. Daxx also has been reported to interact with histone deacetylases [66] that in turn associate with the lamina. 

While the finding of other possible chromatin stabilizing associations on lamin A could explain the failure to observe an increase in chromosome mobility with the histone-binding site antibody injections, at the same time, our study has potentially identified a more specific and new function for the specific lamin A histone-binding site. The combination of a delay in DNA replication and failure of mitotic entry upon microinjection of the histone-binding site antibodies may indicate a novel sensor for complete DNA replication that serves as a new mitotic entry “checkpoint”. While this is purely speculation at this point, it is consistent with ever-increasing evidence that the nuclear periphery and spatial genome organization are important for DNA replication [67,68]. It would be very exciting if antibody blocking of lamin-histone interactions removed stabilizing forces on the late-replicating peripheral heterochromatin and resulted in either defective replication or loss of biophysical tension that could be sensed by the cell to block entry into mitosis. Unfortunately, this would be very difficult to prove on individual microinjected cells and, thus, would require the development of additional reagents, such as nanobodies, that could be stably and inducibly expressed in a population. The idea of a biophysical tension sensor is also interesting as mitotic chromatin condensation has been observed to typically start at the nuclear periphery and in *Drosophila* was shown to be close to centromeres and telomeres [69]. This is particularly interesting in light of the association of CenpB with the A∆ structures. At the same time, it is important to remember that there are other possible explanations for the effects observed. Although obtaining the same effects with Fab fragments indicates that the failure to enter mitosis is not due to lamin crosslinking, the mapped histone-binding site overlaps with phosphorylation sites that are modified in mitosis. Nonetheless, these sites are also modified in interphase, and none of the sites was directly shown to be critical for mitotic lamina disassembly overlap with the region targeted by the antibodies [70]. It is also possible that the replication defect reflects an earlier more internal effect of the antibodies as lamin A has both peripheral and nucleoplasmic pools.

Together, these results argue that answering the question of how nuclear envelope tethering contributes to genome stability, mobility and organization will be even more difficult to address experimentally in a specific manner due to the redundancy of interactions. The accumulating evidence of both general and specific chromatin interactions for nuclear envelope transmembrane proteins [45,47,71,72] further complicates such experiments. Nonetheless, the findings presented here make clear that understanding the many functions of lamins in both normal and disease states will require a much more careful mapping of specific lamin-chromatin interactions than has been undertaken to date. The indications of so many different direct or indirect interactions for lamin A together with indications of potentially distinct chromatin specificities for each lamin subtype (Figure 1b) argues that the field further needs to investigate the specificity of lamin-chromatin interactions for all lamin subtypes in parallel.

## Abbreviations

PML	promyelocytic leukaemia protein
HP1	heterochromatin protein 1
